# Morphology, Optical Properties and Photocatalytic Activity of Photo- and Plasma-Deposited Au and Au/Ag Core/Shell Nanoparticles on Titania Layers

**DOI:** 10.3390/nano8070502

**Published:** 2018-07-06

**Authors:** Alexander Müller, Sandra Peglow, Michael Karnahl, Angela Kruth, Henrik Junge, Volker Brüser, Christina Scheu

**Affiliations:** 1 Max-Planck-Institut für Eisenforschung GmbH (MPIE), Max-Planck-Straße 1, 40237 Düsseldorf, Germany; amueller@lbl.gov; 2 Department of Chemistry and Center for NanoScience (CeNS), University of Munich (LMU), Butenandtstrasse 5-13, 81377 Munich, Germany; 3 Leibniz Institute for Plasma Science and Technology (INP), Felix-Hausdorff-Straße 2, 17489 Greifswald, Germany; sandra.peglow@inp-greifswald.de (S.P.); angela.kruth@inp-greifswald.de (A.K.); brueser@inp-greifswald.de (V.B.); 4 Leibniz Institute for Catalysis at the University of Rostock (LIKAT), Albert-Einstein-Straße 29a, 18059 Rostock, Germany; michael.karnahl@oc.uni-stuttgart.de (M.K.); henrik.junge@catalysis.de (H.J.)

**Keywords:** noble metal nanoparticles, core-shell structures, photodeposition, magnetron sputtering, photocatalysis, hydrogen production, localized surface plasmon resonance, structure-property relationships

## Abstract

Titania is a promising material for numerous photocatalytic reactions such as water splitting and the degradation of organic compounds (e.g., methanol, phenol). Its catalytic performance can be significantly increased by the addition of co-catalysts. In this study, Au and Au/Ag nanoparticles were deposited onto mesoporous titania thin films using photo-deposition (Au) and magnetron-sputtering (Au and Au/Ag). All samples underwent comprehensive structural characterization by grazing incidence X-ray diffraction (XRD), scanning electron microscopy (SEM), and transmission electron microscopy (TEM). Nanoparticle distributions and nanoparticle size distributions were correlated to the deposition methods. Light absorption measurements showed features related to diffuse scattering, the band gap of titania and the local surface plasmon resonance of the noble metal nanoparticles. Further, the photocatalytic activities were measured using methanol as a hole scavenger. All nanoparticle-decorated thin films showed significant performance increases in hydrogen evolution under UV illumination compared to pure titania, with an evolution rate of up to 372 μL H_2_ h^−1^ cm^−2^ representing a promising approximately 12-fold increase compared to pure titania.

## 1. Introduction

For decades, mankind has relied heavily on unsustainable energy sources such as coal, oil, gas or nuclear power [1,2,3]. In recent years, concerns over climate change, environmental pollution, resource depletion, and safety issues have led to global efforts towards the development of sustainable and clean energy sources [1,2,3,4,5], with some of the most promising approaches making use of the almost unlimited energy of the sun [2,6,7,8,9,10,11,12]. One of these approaches is photocatalytic water splitting, where solar energy is used to photogenerate charge carriers in a semiconductor. The excited electrons then reduce protons, producing “green” hydrogen, which can be stored, transported, and finally converted back to energy using fuel cells. This approach therefore opens a promising route towards a carbon-neutral energy landscape.

Titania in the rutile phase was the first material found to have conduction and valence band positions suitable for photocatalytic water splitting [13,14,15]. It also has several other desirable properties such as high corrosion-resistance, abundance, low price, and non-toxicity [16]. Therefore, titania, both in the rutile and in the low-temperature anatase modification, is still one of the most studied materials for water splitting and other photocatalytic applications like the degradation of organic compounds [5,15,17,18,19]. Its biggest drawback is the comparatively large band gaps of 3.2 eV for anatase and of 3.0 eV for rutile, corresponding to absorption edges at wavelengths of 386 and 416 nm, respectively [13,16,17,20]. Accordingly, significant light absorption and photogeneration of charge carriers can only occur within or near the UV region, which accounts for merely 5% of the total energy of the solar spectrum [21,22]. This limits the theoretical maximum efficiency to 1.3% for anatase and 2.2% for rutile [17]. As the performance strongly depends on the band gap, it can be enhanced by either introducing additional electronic states into the band gap via doping and/or by depositing a second light-absorbing material that absorbs within the visible region and acts as a photosensitizer for titania [21,23,24]. Popular photosensitizers, that are also used in solar cells, are (metal) organic dyes [25,26,27,28,29,30]. They are, however, often unstable under UV radiation and in the chemical environment present during water splitting [25].

More suitable for this application are nanoparticles based on noble metals such as Au, Ag or Cu. In these metals, light can induce a localized collective electron oscillation, a so-called “plasmon”, near the nanoparticle surface [22,23,31,32]. The energy of this surface plasmon strongly depends on the size, shape and dielectric constant of the nanoparticle as well as of the surrounding medium [22,23,32,33]. By optimizing these parameters, the wavelength of the localized surface plasmon resonance (LSPR) can be shifted to the desired range, allowing for visible or even near-infrared light absorption [22,23,32,33,34,35]. These plasmon-induced electrons can transfer to an electron-acceptor such as titania [24], increasing visible light-induced charge separation and enhancing the efficiency of photoreactions. If the LSPR frequency is at or near the semiconductor band gap, the electromagnetic field stemming from the plasmon excitation can lead to charge carrier formation in the supporting semiconductor [36,37,38]. Under these conditions, the large scattering cross section of the plasmon oscillation in noble metal nanoparticles enhances the optical pathway of the incident photons leading to increased light absorption [39]. In addition to their function as photosensitizer, noble metal nanoparticles can enhance the performance of the semiconductor as co-catalysts by providing chemically active sites with low activation barriers [40], prolonging charge carrier lifetime [40], and serve as a reservoir for electrons generated in the titania by UV light [24,41,42,43].

Several studies on photosensitizing titania using noble metal nanoparticles were carried out on suspended powders [40,42,44,45,46,47,48]. In contrast, we deposited Au-based nanoparticles onto anatase thin films. Compared to powdered samples, immobilizing the catalyst allows for precise control over morphology, light absorption and catalytic behavior, and therefore for high reproducibility of the measured photocatalytic performance [49,50]. The samples are also easier to handle, and agglomeration of particles is not an issue. The performance typically depends greatly on the morphology of the sample, and studying the correlation of synthesis conditions, morphology and properties is of paramount importance. In this study, two different methods were used to deposit metallic nanoparticles. First, gold nanoparticles were prepared by in situ photodeposition, which is a widely established approach that works well at the laboratory scale [51]. Second, both gold and silver/gold nanoparticles, which often show significantly increased catalytic activity compared to the respective monometallic nanoparticles [52,53], were deposited by a radio frequency (RF) magnetron sputtering process followed by an annealing step [54,55]. In comparison to photodeposition, physical deposition processes such as this one can often be scaled up to an industrial scale. Care was taken that the noble-metal loading of the two Au-TiO_2_ samples was comparable. The crystal structures of all phases were confirmed via grazing incidence X-ray diffraction, and the morphologies of the samples were analyzed using scanning and transmission electron microscopy. As expected, both synthesis methods resulted in considerably different particle distributions, particle size distributions and defect structures. We further measured the UV-vis spectra and the photocatalytic evolution of hydrogen from water using the sacrificial electron donor methanol. Methanol was chosen as past studies had shown it to be an excellent hole scavenger, allowing us to neglect surface kinetics in the discussion and simplifying the complex behavior of this system [56,57,58]. All results were correlated to each other, and we hope that our combined findings contribute to an in-depth understanding of the interplay between synthesis, structure, and properties.

## 2. Materials and Methods

Titania films were deposited onto fluorine-doped tin oxide substrates (fluorine doped tin oxide (FTO), TCO 22-7, Solaronix, 25 × 25 mm, Aubonne, Switzerland) by a direct current (DC) reactive magnetron sputtering process previously described by Kruth et al. [54]. The cylindrical Ti target (Ti-133, Bekaert Advanced Coatings NV, Deinze, Belgium, 135 mm diameter, 58.5 mm length) was sputter-cleaned in an Ar atmosphere at 8 kW for 5 min. After stabilizing the process conditions in an O_2_/N_2_/Ar atmosphere (6 standard cm^3^/min (sccm) O_2_, 3 sccm N_2_, and 60 sccm Ar) at 3 Pa for 8 min, TiO_2_ was plasma-deposited at a magnetron power of about 5.3 kW and a magnetron voltage of 450 V. To transform the resulting amorphous TiO_2_ into anatase, the samples were annealed for 1 h at 400 °C with a heating rate of 10 °C/min in an oxygen atmosphere at a flow rate of 0.05 standard L/min (slm).

Au and Au/Ag core-shell nanoparticles were deposited onto the titania films described above using a RF-magnetron sputtering process previously published by Peglow et al. [55]. Au and Ag sputtering targets (both of 3 mm thickness, two inch diameter and purity of 99.999%, MaTeck, Juelich, Germany, were placed at respective distances of 9.5 and 5.5 cm from the substrate. Small sputtering rates were achieved by shielding the magnetic field with a 1 mm thick iron disk (99.95%, MaTeck) placed between the magnetron and the two targets. The deposition was performed at a magnetron power of 50 W at a working pressure of 5 Pa in an argon atmosphere (15 sccm gas flow). After each deposition, the samples were annealed by placing them in a quartz tube that was inserted into a tube furnace (Zirox GmbH, Greifswald, Germany, kept at 400 °C by a thermal controller (Eurotherm 2416, Limburg an der Lahn, Germany) for 30 min. The O_2_ atmosphere (0.05 slm) was regulated using a gas flow controller (MKS Instruments Multi Gas Controller 647B, Andover, MA, USA). Au nanoparticles were synthesized by depositing gold over a period of 300 s, resulting in a nominal layer thickness of (6.6 ± 0.7) nm. Deposition was followed by an annealing step, a second, 300 s long deposition step and a final annealing step leading to an estimated total layer thickness of (13.2 ± 1.5) nm. To obtain Au/Ag core-shell nanoparticles, Au-deposition for 188 s resulted in a nominal layer thickness of (4.1 ± 0.5) nm and was followed by Ag-deposition over 36 s, resulting in a nominal layer thickness of (2.5 ± 0.1) nm, and one final annealing step.

A second series of Au nanoparticles was prepared by in situ photodeposition onto titania films following a synthesis procedure described by Gärtner et al. [40]. The temperature of a double-walled reaction vessel was adjusted to 25 °C by a thermostat. The titania film (25 × 25 mm) was inserted into the reactor with a glass holder. Subsequently, the gold precursor (NaAuCl_4_·2 H_2_O, 3.1 mg) was added. The whole system was evacuated and flushed with argon to remove any other gases. Then, 40 mL freshly distilled water and 40 mL methanol were added under argon counter flow, resulting in a final concentration of the gold precursor of about 0.1 mmol/L. The photodeposition was initiated with a Hg-lamp (7.2 W output, Lumatec Superlite 400, Deisenhofen, Germany) equipped with a 320–500 nm filter [40]. A swift color change from light yellow to dark red occurred, with the formed hydrogen escaping by a bubbler. The reaction was stopped after 3 h and the sample was washed with deionized water and ethanol prior to drying in air.

Phase identity and average crystallite sizes were determined by grazing incidence X-ray diffractometry (GIXRD). Diffractograms were obtained using a Bruker D8 Advance (Billerica, MA, USA) with a Cu-Kα source. The measurement was carried out in a 2θ-range of 20°–80°at an incident angle of 0.5°, with a step width of 0.02° and a measurement time of 5 s per step. Crystallite sizes were calculated from the (200) reflection of Au as well as the (200) and (101) reflections of anatase using a combination of the Stokes–Wilson and the Variance model and fitting the correlated integral widths by a Pearson VII function [59].

Scanning electron microscopy (SEM) was performed on a JEOL JSM 7500F (Tokyo, Japan) with a field emission gun, a semi-in-lens conical objective lens, and a secondary electron in-lens detector. At an acceleration voltage of 15 keV, a resolution of 1.0 nm was achieved.

A comprehensive structural analysis was carried out using a FEI Titan 80-300 transmission electron microscope (TEM, Hillosboro, OR, USA). Bright-field (BF) and high-resolution TEM (HRTEM) images were recorded on a Gatan UltraScan 1000 CCD (Pleasanton, CA, USA), scanning TEM (STEM) images with a Fischione Model 3000 high angle annular dark-field (HAADF) detector (Export, PA, USA) and energy-dispersive X-ray (EDX) spectra with an EDAX detector (Mahwah, NJ, USA). Samples were prepared by either scratching material of the substrate and depositing it onto a TEM grid with a holey carbon film or by preparing a cross-section according to a procedure adapted from Strecker et al. so that the sample was prepared at room temperature [60].

The optical properties of the different samples were investigated using a PerkinElmer Lambda UV-vis 850 spectrophotometer with a L6020322 150 mm integrating sphere and a Spectralon Reflectance Standard (>99% R, USRS-99-020, PerkinElmer Inc., Waltham, MA, USA). The UV-vis spectra were recorded by measuring the diffuse transmission at wavelengths from 250 nm to 850 nm. Calculation of the absorbance A was carried out under the assumption that no reflection occurs at the sample using Equation (1) [54,61],

A = −log10(*I*_T_/*I*_0_), (1)

where A is the absorbance in arbitrary units, *I*_T_ is the measured transmission intensity in percent and *I*_0_ is the incident light intensity, which equals 100%.

Photocatalytic hydrogen evolution experiments were performed under argon atmosphere and the strict exclusion of oxygen using freshly distilled and degassed solvents. The sample was introduced into a double-walled, thermostatically-controlled reaction vessel by a glass holder and aligned in parallel to the planar optical window. This setup allowed for a reproducible experimental arrangement and a direct illumination of the sample without blocking by the cooling water. Furthermore, a complete irradiation of the 25 × 25 mm thin film layer was ensured. Subsequently, the photoreactor was connected to an automatic gas burette and repeatedly evacuated and filled with argon in order to exclude any oxygen. Then, the solvent mixture (80 mL), composed of water and methanol in a ratio of 1/1 (*v*/*v*), was added, fully covering the layer. The temperature of the whole system was maintained at 25 °C by a thermostat. After stirring for at least 10 min at 300 rounds per minute to reach thermal equilibrium, the reaction was started by switching on a Hg-lamp (Lumatec Superlite 400, Deisenhofen, Germany) equipped with either a 320–500 nm or a 400–700 nm filter. In both cases, the light intensity was set to 7.2 W. The amount of evolved gases was continuously monitored by the automatic gas burette, while the gas composition was analyzed by gas chromatography. A more detailed description of the experimental setup can be found in the literature [40].

## 3. Results and Discussion

### 3.1. Structural and Morphological Characterization

**Figure 1:**
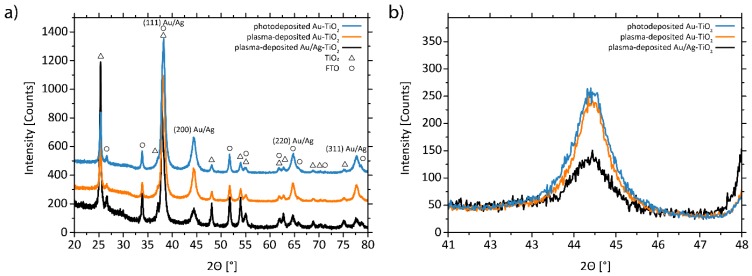
(**a**) Grazing incidence X-ray (GIXR) diffractograms of the three noble metal-decorated samples and (**b**) close-up of the Au/Ag (200) peaks. The intensities of the curves in (**a**) were shifted vertically by constant factors, as they would overlap otherwise.

GIXR diffractograms (Figure 1a) indicate the phase identities of all samples [62,63,64]. Annealing of the titania films led to crystallization of the material in the anatase modification, with an average crystallite size of approximately 25 nm. This is in agreement with earlier results [27,54]. Monometallic Au and bimetallic Au/Ag nanoparticles crystallize in the face-centered cubic Fm-3m symmetry (space group 225), with the lattice parameters of Ag and Au being too similar to differentiate between the two phases. The (200) peaks of Au and Au/Ag did not show any overlap with those of other phases and were used for further analysis (Figure 1b). The intensities of the (200) peaks of Au in photodeposited and plasma-deposited Au-TiO_2_ are similar, indicating similar metal loading. In contrast, a weaker peak is observed for plasma-deposited Au/Ag-TiO_2_, explicable by the lower metal loading. Calculated average crystallite sizes of all three samples were comparable at around 6–7 nm, with no drop for the bimetallic sample with significantly lower metal loading. It should, however, be considered that crystallite size calculations from XRD are limited to average values for crystalline domains and only assume the presence of ideal, spherical crystallites.

**Figure 2:**
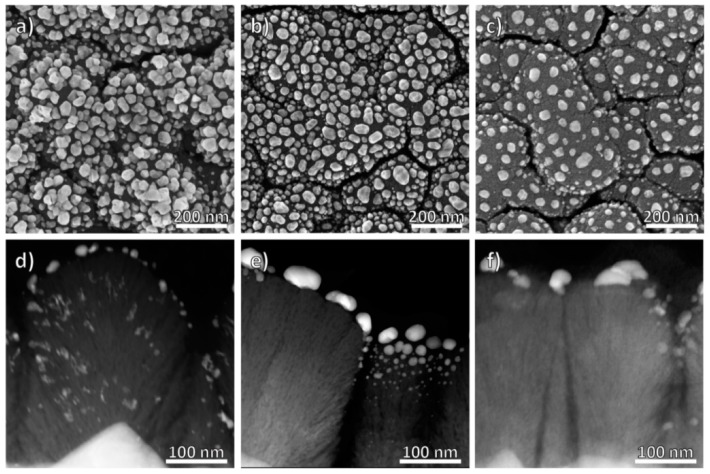
Top-view SEM (scanning electron microscopy) and cross-section scanning transmission electron microscopy (STEM) images of photodeposited Au-TiO_2_ (**a**,**d**), plasma-deposited Au-TiO_2_ (**b**,**e**) and plasma-deposited Au/Ag-TiO_2_ films (**c**,**f**).

In Figure 2, electron microscopy images of the three samples are shown. Top-view images were acquired by scanning electron microscope (SEM) (Figure 2a–c), cross-sections by HAADF-STEM (high angle annular dark-field cross-section scanning transmission electron microscopy) (Figure 2d–f).

The titania layer is polycrystalline, approximately 300 nm thick and composed of individual pillars, each of them grown on top of a FTO pyramid. This microstructure is typical for ZI thin film growth [65,66]. The fibrous titania pillars are in the anatase modification, with 3–5 nm wide pores elongated in the direction perpendicular to the TiO_2_/FTO interface. The porosity of the titania was quantified from HAADF-STEM images. The signal intensity I in such images scales with the mean atomic number Z raised by an exponent *y* (Equation (2)) [67]. As the FTO and the underlying SiO_2_ substrate are compact layers, y can be calculated. This calculation, as well as those following, were done using several hundred acquisition points for each material and working with average values. The mean atomic number of the titania layer is given by:

(2) $$\frac{I_{\mathrm{Titania}}}{I_{\mathrm{FTO}}}={\left(\frac{Z_{\mathrm{Titania}}}{Z_{\mathrm{FTO}}}\right)}^{y}\to Z_{\mathrm{Titania}}{=Z}_{\mathrm{FTO}}\cdot \sqrt[{y}]{\frac{I_{\mathrm{Titania}}}{I_{\mathrm{FTO}}}},$$

The porosity is then equal to the ratio of the mean atomic numbers of the measured, porous and the theoretical, compact layer and was determined as ~10%, indicating low porosity.

The photodeposited Au nanoparticles are found both on top of the columns and incorporated into pores of the TiO_2_ layer. The latter indicates that some of the pores are open at the surface and can be filled with the gold precursor solution during the photodeposition process. In contrast, RF-sputtered Au and Au/Ag nanoparticles are found on top of the titania layer which is typical for such sputter deposition processes. The particles located in the cavities are significantly smaller than the grains formed on top of the columns (Figure 2). Compared to plasma-deposited Au-TiO_2_, approximately half of the nominal layer thickness was deposited during the synthesis of plasma-deposited Au/Ag-TiO_2_ (Figure 2b,c). This reduction leads to a sparser distribution of nanoparticles of roughly the same size. The half-as-high loading was also confirmed by EDX measurements (Table 1). The noble metal content could be determined by calculating the mass of the TiO_2_ layer from the thickness and the density and comparing it with the ratio of noble metals to Ti. In contrast to Au/Ag-TiO_2_, the Au-loading in photodeposited Au-TiO_2_ and plasma-deposited Au-TiO_2_ is very similar.

**Figure 3:**
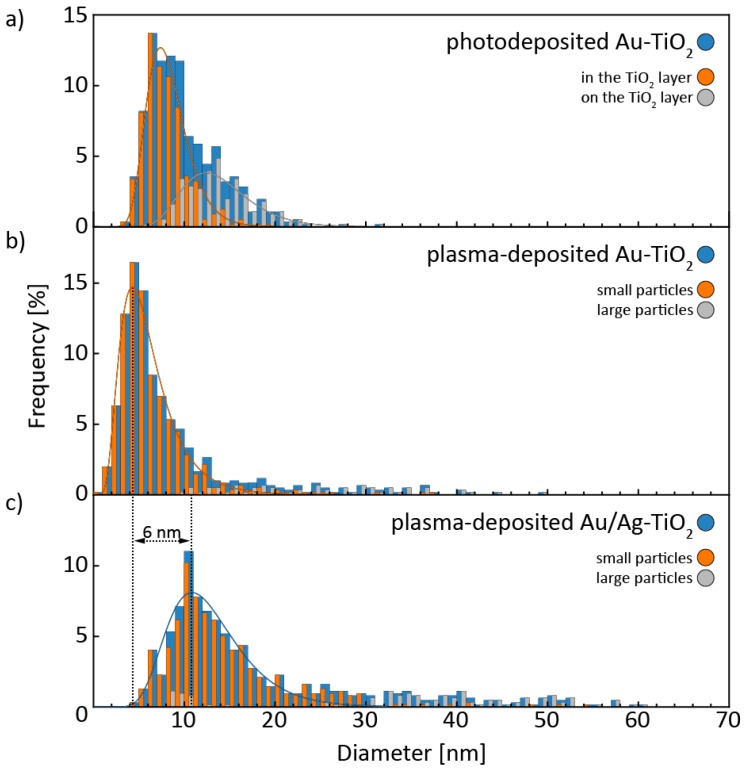
Size distributions of the noble metal nanoparticles in (**a**) photodeposited Au-TiO_2_, (**b**) plasma-deposited Au-TiO_2_ and (**c**) plasma-deposited Ag/Au-TiO_2_. All size distributions were split into two sub-distributions each. As explained in the main text, “large particles” refers to particles we believe have coarsened, “small particles” to those that have not. Please note that the frequency values only apply to the size distribution of the whole sample, but not to the sub-distributions.

In Figure 3, the size distributions of all three samples are shown. As already mentioned, photodeposited Au nanoparticles grew both inside and on top of the titania layer, which is reflected by the two different log-normal size distributions used to describe the experimentally determined size distribution. Photodeposited Au nanoparticles inside the titania layer, which account for 66% of all Au nanoparticles, have a different size distribution than those found on top of the titania layer (Figure 3a). However, a log-normal distribution, which has previously been applied to the size distributions of Au nanoparticles synthesized by several, solution-based synthesis procedures, could be used to describe both [68]. The size distribution of Au nanoparticles inside the titania is shifted towards smaller diameters, indicating that the growth is slowed down or stopped within the pores of the TiO_2_ layer.

To interpret the size distributions of plasma-deposited particles (Figure 3b,c), two underlying processes, deposition and annealing, have to be considered. Previous studies have shown that sputter deposition produces thin films, which dewet during annealing [55,69,70]. Some of these isolated particles then grow via a coarsening mechanism. This coarsening step is expected to depend strongly on a low surface roughness to prevent particle pinning and facilitate particle diffusion. We used these assumptions to split each size distribution in two by considering large nanoparticles on top of smooth TiO_2_ surfaces as resulting from a coarsening mechanism. With this assumption, the non-coarsened particles, which account for 86% in plasma-deposited Au-TiO_2_ and 83% in plasma-deposited Au/Ag-TiO_2_, can be fit very well to a log-normal distribution. Attempts to model the other particle fraction with a size distribution failed due to their relative scarcity. Compared to pure plasma-deposited Au nanoparticles, the maximum of the log-normal distribution of the smaller, non-coarsened Au/Ag nanoparticles is shifted from 4 to 10 nm (Figure 3b,c). In spite of the reduced nominal layer thickness, the increase in size indicates that the initial deposition of Au directs their size, and not the subsequent Ag deposition or the annealing step. This hints at the observed shift resulting from Ag being added to a pre-existing Au nanostructure. Of course, this argument only applies to non-coarsened, small nanoparticles.

**Figure 4:**
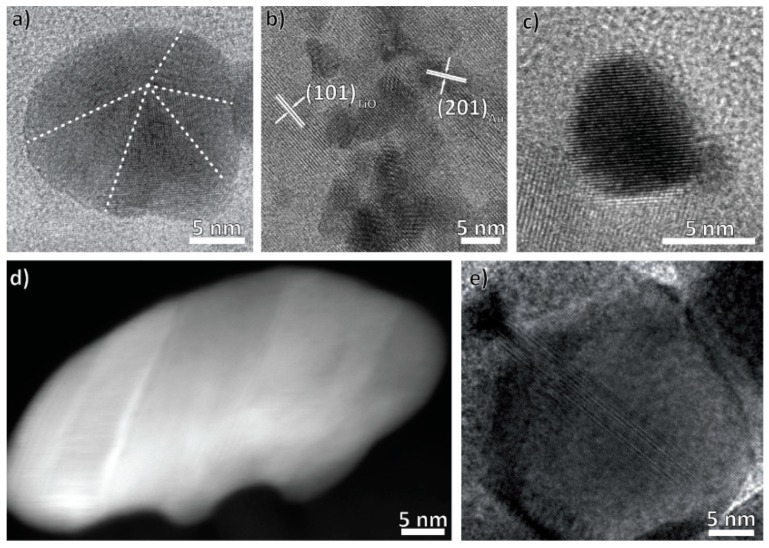
Representative images of different defect structures of the noble metal nanoparticles. In (**a**) and (**b**) high-resolution TEM (HRTEM) images of the photodeposited Au nanoparticles are shown: (**a**) is a five-fold twinned particle on top of the titania and (**b**) defect-free nanoparticles inside the titania. (**c**–**e**) show plasma-deposited nanoparticles: (**c**) is representative for small, defect free nanoparticles, (**d**) of those with stacking faults and (**e**) of those with grain boundaries.

The nanoparticles can possess several different defect structures (Figure 4). The photodeposited Au nanoparticles can be inside and outside of the titania layer, with each fraction having its own predominant defect structure. Photodeposited nanoparticles on top of the titania are predominantly five-fold twinned (Figure 4a), with few occurrences of other defect structures such as grain boundaries. Such a twinning is energetically favorable for small nanoparticles and therefore very common [71,72,73,74,75]. In contrast, all particles observed within the titania layer were monocrystalline (Figure 4b). However, a definite correlation of nanoparticle size and defect structure could not be concluded. The existence of defects not inherent to the metal or of an oxide surface layer large enough to form a defined crystal structure could be excluded from HRTEM images.

For the plasma-deposited nanoparticles, single-crystallinity, five-fold twinning, stacking faults, and grain boundaries were observed (Figure 4d,e). As with photodeposited nanoparticles, we could not conclude a correlation of size and defect structure, with the exception of grain boundaries, which were very common in big nanoparticles. We tentatively ascribe these to the coarsening process. These particles also often have little protrusions that fill nooks in the titania substrate. Once again, other defects can be excluded from HRTEM images.

**Figure 5:**
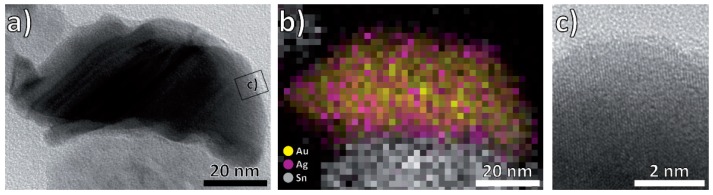
(**a**) BF (Bright-field) images of a representative Au/Ag nanoparticle; (**b**) energy dispersive X-ray (EDX) map of the same particle, showing an accumulation of Ag at the surface; (**c**) HRTEM image of the nanoparticle surface.

Bimetallic Au/Ag nanoparticles could potentially be alloyed or form core-shell nanoparticles [76,77]. The melting temperatures of Ag and Au decreases with the nanoparticle size, but are always high compared to the highest temperature reached during synthesis (400 °C) [78,79,80]. Alloying therefore seems unlikely. In accordance, EDX maps confirmed the formation of a uniform, 2–3 nm thick Ag shell around the Au core. As both metals crystallize in the face-centered cubic structure and their lattice parameters differ by only 0.2%, we observe defect-free continuation of the crystal structure of Au by Ag without any phase boundary (Figure 5c) [81]. Previous studies have shown inhomogeneous deposition of gold and silver, and the core-shell nanoparticles presumably result from nanoparticle attachment during the annealing treatment [82,83]. Interestingly, former experiments by one of the co-authors, in which the Ag/Au deposition order was reversed, also yielded Au/Ag-core-shell nanoparticles [55]. The deposition order can therefore not be the decisive factor when determining which metal becomes the core and which the shell. Unfortunately, growth mechanism studies so far mostly focus on wet-chemical synthesis methods and do not apply to our synthesis method [82,83]. Looking at the thermodynamics of the two possible core-shell configurations, four different energies contribute to the total energy: those of bulk Au and bulk Ag, the interface energy of the interface between Ag and Au, and the surface energy of the shell material. Assuming the amount of bulk material is the same in both possible configuration, the volumes and therefore the enthalpies associated with the interface and the two bulk phases are identical for both and only the contribution of the surface changes when exchanging core and shell material. As the surface energy of Au is approximately 40% higher than that of Ag, the total enthalpy is reduced by forming a silver instead of a gold shell. We assume this to be the dominant driving force for the preferred creation of Au/Ag core-shell nanoparticles over Ag/Au core-shell nanoparticles [84].

### 3.2. Optical Properties

**Figure 6:**
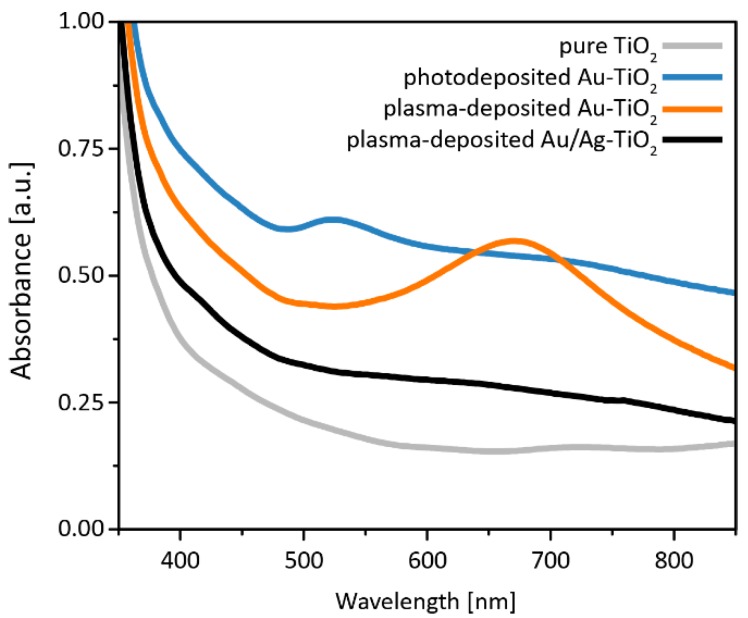
UV-vis spectra showing the absorbance of all samples.

Figure 6 shows the absorbance spectra of the Au, Au/Ag and pure TiO_2_ samples calculated from diffuse transmission measurements using Equation (1). The strong absorbance at wavelengths shorter than approximately 400 nm corresponds to the anatase band gap of 3.2 eV [13,16,17,20]. Throughout the measurement range, the absorbance of the samples never reach an intensity of zero, which is attributed to Rayleigh scattering at crystalline domains as well as pores of the TiO_2_ layer, with the noble metal nanoparticles acting as additional scattering sites [85,86,87,88]. Consequently, the intensities of this background absorption can be correlated to nanoparticle loading and distribution of the different samples. Nanoparticle-decoration leads to stronger absorbance than pure TiO_2_, with photodeposited Au-TiO_2_ reaching the highest background absorbance, followed by plasma deposited Au-TiO_2_ and then plasma-deposited Au/Ag-TiO_2_. The total noble metal mass in Au/Ag-TiO_2_ is roughly half that of both Au-TiO_2_ samples (Table 1), and it consequently absorbs less than those two. The high absorbance of photodeposited Au-TiO_2_ could be attributed to the dense coverage of the titania surface (Figure 2a) and the additional presence of nanoparticles embedded in TiO_2_ pores (Figure 2d).

Both plasma- and photodeposited Au-TiO_2_ have additional absorption bands at 550–800 nm and 480–580 nm, respectively, caused by the excitation of localized surface plasmon resonances (LSPR). The positions and shapes of these bands are determined by the particle shape [89], the contact area with the titania [90], the size [91], and the size distribution [92]. An overlap of these factors hinders the interpretation of polydispersed particle ensembles. The center of the absorption band of photodeposited Au-TiO_2_ matches the LSPR frequency of 520 nm previously described for isolated spherical gold nanoparticles [93,94]. TEM images confirm the existence of such particles (Figure 4a), however, many irregularly shaped nanoparticles with diameters more than 100 nm are found in top view SEM micrographs (Figure 2a). The intense band of plasma-deposited Au-TiO_2_, however, is red-shifted. This could be explained by the non-spherical shape of the large Au nanoparticles [39,94,95,96], plasmon-coupling [22,94,95,97,98], and/or a large contact area with the TiO_2_ [97] as suggested by SEM and TEM micrographs (Figure 2 and Figure 4). Although embedded nanoparticles were not found in plasma-deposited samples, the particles adapt to the titania surface (Figure 4 and Figure 5). The band broadening could result from large nanoparticle size and/or shape distributions. No distinct bands were observed for plasma-deposited Au/Ag-TiO_2_, even though two would be expected: one stemming from the outer shell surface, and one from the Au/Ag interface [99,100,101]. A possible explanation is the significantly lower metal loading than the two Au-TiO_2_ samples, which should lead to weaker plasmon bands. Furthermore, the peak related to the outer silver shell (which would be expected at around 400 nm) could overlap with the absorbance band of the TiO_2_ substrate [99].

### 3.3. Catalytic Properties

**Figure 7:**
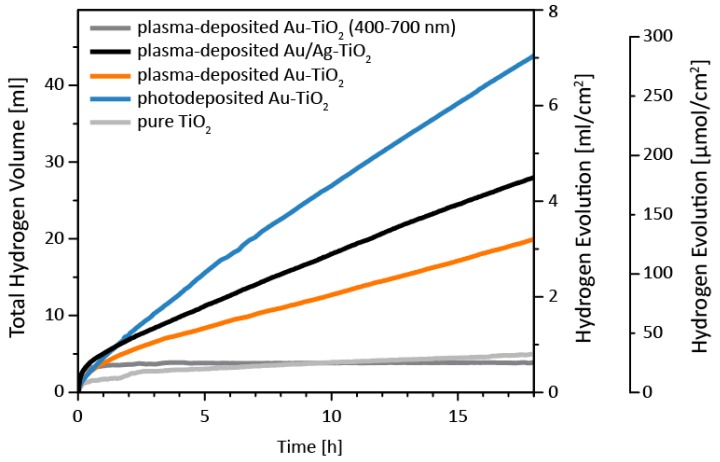
Hydrogen evolution of all samples from a methanol/water mixture. Conditions: 80 mL MeOH/H_2_O (1/1, *v*/*v*), Lumatec Hg-light source equipped with a 320–500 nm filter, 7.2 W output, 25 °C. In addition, the H_2_ evolution of plasma-deposited Au-TiO_2_, acquired using a 400–700 nm filter, is plotted exemplarily (dark grey). Measurements of all samples under the same visible light illumination yielded perfectly overlapping curves and we therefore chose to present only the measurement of the sample with the strongest localized surface plasmon resonances (LSPR) band.

The photocatalytic performance of the different layers to reduce protons to molecular hydrogen was studied under visible light illumination (400–700 nm) and under UV-vis illumination (320–500 nm) with methanol as a hole scavenger (Figure 7, Table 2).

All hydrogen evolution curves (Figure 7) show a strong initial increase during the first minutes, followed by a smaller, but constant hydrogen evolution rate. The steep rise at the beginning of each measurement is caused by an increase in pressure in the automatic gas burette due to heating of the photoreactor upon irradiation with UV-vis light. Within an hour, thermal equilibrium is reached by external cooling with a thermostat (25 °C). Therefore, the later, constant region is more representative of the catalytic activity and the first hour of each measurement was disregarded when discussing the curves (Figure 7) or determining the hydrogen evolution rates presented in Table 2. No further loss in activity could be observed for up to 18 h, indicating stable operation.

Under visible light illumination, the hydrogen evolution rate was zero and no hydrogen was measured apart from the initial rise (Figure 7). In this regime, light is absorbed in the noble metal nanoparticles via surface plasmon resonance, and then electrons are injected into TiO_2_ [42,44]. Given this mechanism and the UV-vis spectra (Figure 6), which show only weak bands attributed to plasmon formation, the weak hydrogen evolution can be explained.

Under UV illumination, only TiO_2_ absorbs light and the noble metal nanoparticles act as co-catalysts (Figure 6) [40,41,42,44,45] by providing chemically active sites with low activation barriers [40], prolonging charge carrier lifetime [40], and by serving as an electron reservoir [24,41,42,43]. Previous photocatalytic experiments under similar conditions revealed a drastic increase in H_2_ evolution upon deposition of noble metal nanoparticles [40,41,42,44,45]. This is confirmed in our experiments, with all samples having a significantly increased hydrogen evolution rate compared to pure TiO_2_.

As mentioned above, all samples onto which noble metal nanoparticles were deposited outperform pure TiO_2_. The largest hydrogen evolution rate of 372 μL h^−1^ cm^−2^ was measured for photodeposited Au-TiO_2_, representing a 12-fold increase. This was followed by plasma-deposited Au/Ag-TiO_2_ (217 μL h^−1^ cm^−2^, 7-fold) and plasma-deposited Au-TiO_2_ (152 μL h^−1^ cm^−2^, 5-fold).

To interpret this order, we first consider the influence of the hole scavenger methanol. Two reactions compete at the surface of the noble metal nanoparticle co-catalysts: the transfer of holes to the electrolyte and the recombination with photogenerated electrons. Adding hole scavengers leads to the transfer reaction being favored over recombination, and the latter is suppressed. Methanol is a very efficient hole scavenger, near-perfect transfer can be assumed [56,57,58], and the kinetics of surface reactions can be neglected. In contrast, light absorption plays an important role and the order in which the samples perform is indeed closely correlated to the amount of light being absorbed in the UV-region. Only plasma-deposited Au/Ag-TiO_2_ is more efficient than expected from light absorption measurements. We attribute this behavior to a reduction of bulk recombination caused by the electric field gradient at the Au/Ag interface, which is known to positively affect the efficiencies of catalytic reactions [52,53].

## 4. Conclusions

Au nanoparticles were deposited onto a mesoporous anatase thin film using two different deposition methods, photo- and plasma-deposition. The second method was further used to prepare bimetallic Au/Ag nanoparticles. Both methods resulted in different particle distributions and particle size distributions. Photodeposited nanoparticles both infiltrated the titania itself and were deposited on top of it, with the latter group of particles being larger on average. In contrast, plasma-deposited nanoparticles were found only at the surface of the titania.

These differences in the morphology could then be correlated to several materials properties. Strong light absorption up to about 400 nm is due to the band gap of titania. Above 400 nm, Rayleigh scattering at crystalline domain boundaries and pores within the TiO_2_ layer, as well as at noble metal nanoparticles, leads to a strong background. Both samples with pure Au nanoparticles further show peaks attributed to LSPR.

The light absorption properties, in turn, strongly influence the photocatalytic performance of the hydrogen evolution reaction from a methanol/water solution under UV illumination. In this wavelength range, Au only acts as a co-catalyst and the performance of all samples but one, plasma-deposited Au/Ag-TiO_2_, was correlated to the light absorption efficiency. The higher-than-expected performance of plasma-deposited Au/Ag-TiO_2_ can be explained by an electric field gradient at the Au/Ag interface, concluding a comprehensive correlation of structure and properties. Consequently, this study shows that a systematic investigation of the interplay of synthesis method, structure and catalytic activity plays an important role in furthering our understanding of such complex systems. We believe that similar studies, along with those on different hole scavenger, surface reaction kinetics, thermal stability etc., will eventually lead to commercial applications and thereby contribute to a sustainable energy mix.

## Figures and Tables

**Table 1:** Noble metal content of the three samples.

Sample	Au-Content (μg/cm^2^)	Ag-Content (μg/cm^2^)
photodeposited Au	17.5 ± 3.7	-
plasma-deposited Au	19.6 ± 6.5	-
plasma-deposited Au/Ag	7.8 ± 3.4	1.4 ± 0.8

**Table 2:** Comparison of the produced amount of hydrogen and the respective photocatalytic activities normalized to the evolution of hydrogen per area and time.

Sample	Total H_2_ Volume (mL)	H_2_ evolution Rate ^a^ (µL h^−1^ cm^−2^)	H_2_ evolution Rate ^b^ (μmol h^−1^ cm^−2^)
pure TiO_2_	5.0	31	1.3
photodeposited Au-TiO_2_	44.1	372	15.2
plasma-deposited Au-TiO_2_	20.0	152	6.2
plasma-deposited Ag/Au-TiO_2_	28.2	217	8.9

^a^ calculated as an average over 17 h, starting after the first hour. ^b^ calculated by applying the van der Waals equation at 25 °C, which yields a molar volume of 24.48 μL/μmol.
